# Radioiodine sorbent selection criteria

**DOI:** 10.3389/fchem.2022.969303

**Published:** 2022-08-31

**Authors:** Brian J. Riley, Krista Carlson

**Affiliations:** ^1^ Pacific Northwest National Laboratory, Richland, WA, United States; ^2^ University of Nevada Reno, Reno, NV, United States

**Keywords:** iodine capture, iodine immobilization, iodine sorbent selection criteria, getter metal, chemisorption

## Abstract

Methods for preventing radioiodine from entering the environment are needed in processes related to nuclear energy and medical isotope production. The development and performance of many different types of sorbents to capture iodine have been reported on for decades; however, there is yet to be a concise overview on the important parameters that should be considered when selecting a material for chemically capturing radioiodine. This paper summarizes several criteria that should be considered when selecting candidate sorbents for implementation into real-world systems. The list of selection criteria discussed are 1) optimal capture performance, 2) kinetics of adsorption, 3) performance under relevant process conditions, 4) properties of the substrate that supports the getter, and 5) environmental stability and disposition pathways for iodine-loaded materials.

## Introduction

**FIGURE 1:**
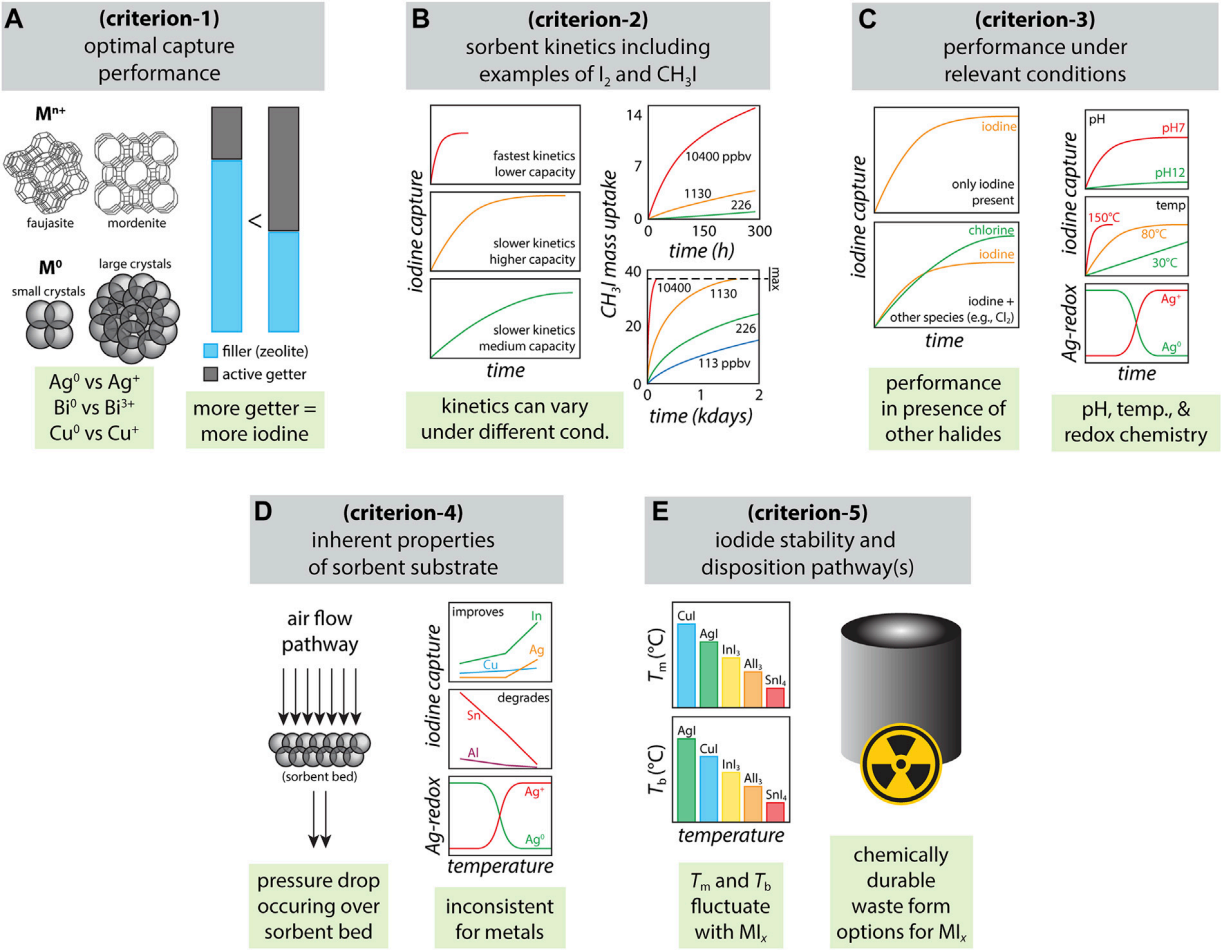
Summary of various parameters for consideration when selecting sorbents for radioiodine: **(A)** optimal capture performance including different types of Ag-zeolites that have different Ag loadings (Riley et al., 2022b) (criterion-1), **(B)** sorbent kinetics including examples based off actual data for I_2_ (Riley et al., 2014) for sulfide-based chalcogels and CH_3_I for Ag^0^-aerogels (Tang et al., 2021) [in parts per billion by volume (ppbv) CH_3_I concentration streams] (criterion-2), **(C)** performance under relevant conditions based off data for I/Cl coadsorption (Matyáš et al., 2021b) and other studies in this area (Riley et al., 2021; Riley et al., 2022a) (criterion-3), **(D)** inherent properties of sorbent substrate (Riley et al., 2021) (criterion-4), and **(E)** iodide stability and disposition pathways (Riley et al., 2021; Reiser et al., 2022) (criterion-5).

Several key parameters need to be considered when selecting a sorbent for capturing radioiodine, which can be relevant for a variety of situations including (a) preventing release of gaseous or ionic iodine species after a nuclear accident into the surrounding environment, (b) capture from the off-gas stream in a molten salt reactor (MSR), (c) capture of gaseous species during reprocessing of used nuclear fuel, and (d) release during medical isotope production. The primary list of sorbent selection criteria includes 1) optimal iodine capture performance of the getter (i.e., loading capacity of chemisorbed iodine under saturated conditions), 2) kinetics of iodine adsorption (i.e., rate of iodine capture), 3) iodine capture performance under relevant process conditions (e.g., pH and temperature of stream, redox sensitivity in oxidizing/reducing environments, iodine selection preference over other components present in the system), 4) properties of the sorbent substrate that supports the getter (e.g., silica aerogel, carbon foam), 5) environmental stability and disposition pathways for iodine-loaded materials. These criteria, graphically shown in Figure 1, are not in any sort of priority order as needs drastically vary with the application. In this paper, these individual parameters will be discussed to provide a perspective on chemisorption-based sorption (compared to physisorption-based sorption) and the areas still in need of study (e.g., radiation stability, capture in a prototypical off-gas stream).

### Optimal iodine capture performance

Stable materials with strong iodine chemisorption are needed for iodine capture to prevent the release of iodine with time or thermal decomposition during heating under prolonged sorbent bed operation as well as during the process to create a waste form (Figure 1A). Once candidate getters have been selected, iodine capture in a saturated environment is performed to understand the maximum loading capacity. Evaluating getter performance in an idealized environment (i.e., no competing or reacting species) is an initial critical assessment to understand the fundamental interactions with iodine before introducing complexity from the process stream.

**FIGURE 2:**
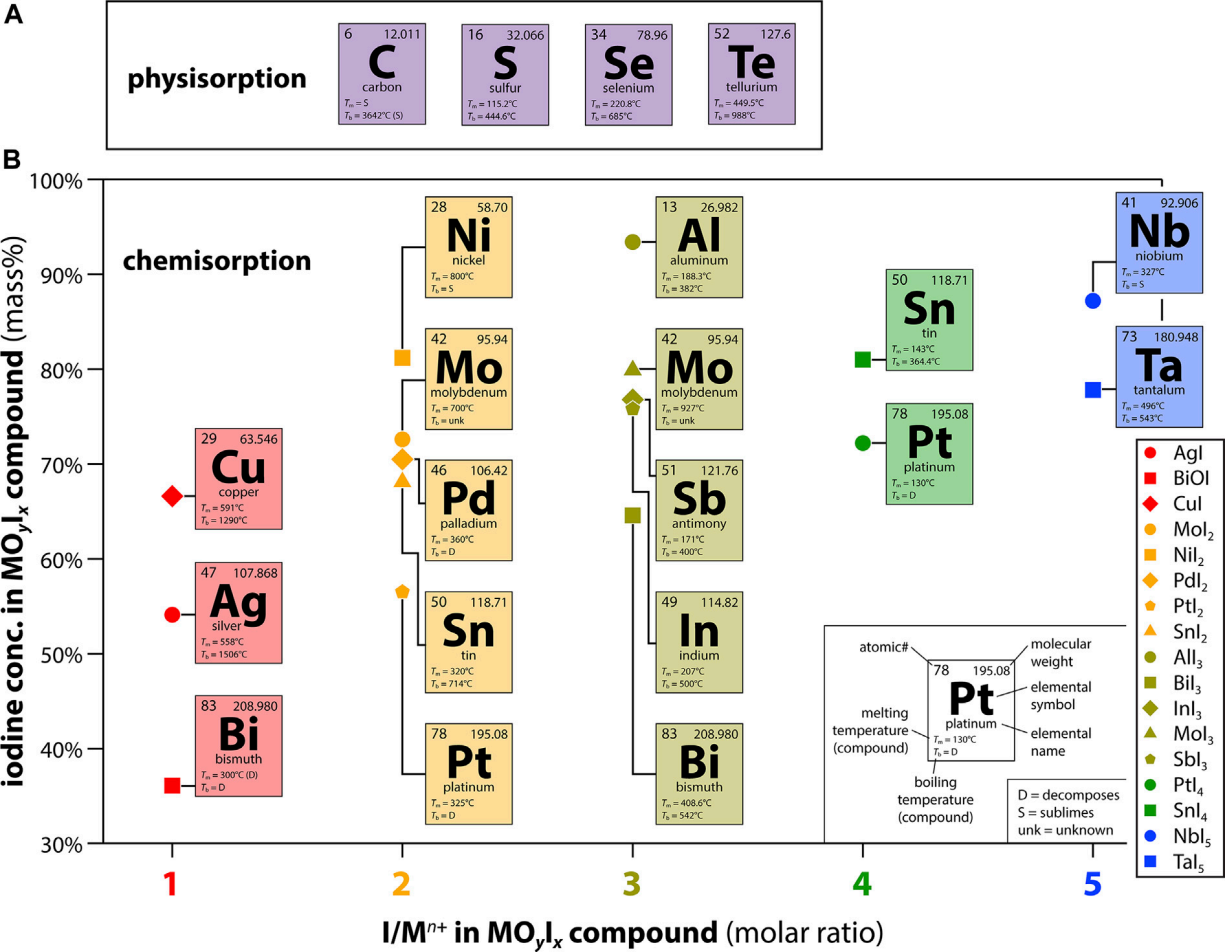
Summary of common species demonstrated in the literature for **(A)** physisorption-based or **(B)** chemisorption-based iodine capture processes. The data in **(B)** show the iodine concentration in the MO_*y*_I_*x*_ compound as a function of the I/M^*n*+^ ratio based on the metal oxidation state in the iodine-loaded complex.

Figure 2 shows some of the elements that have been explored in various forms for the capture of volatile iodine along with the expected MO_*y*_I_*x*_ compounds based on expected metal oxidation states in the iodine-loaded complexes (Maeck et al., 1968; Maeck and Pence 1970; Pence et al., 1970; Pence et al., 1972; Matyáš et al., 2011; Riley et al., 2017; Riley et al., 2020; Matyáš et al., 2021a; Riley et al., 2021; Tesfay Reda et al., 2021). In practice, several of these base metals do not show iodine capture under saturated conditions so specialized conditions might be needed to realize their potential (Riley et al., 2021). Figure 2B provides an overview of the large range in maximum iodine loadings possible by simply selecting different getter metals.

Transition metals and post-transition metals have been extensively evaluated, most commonly in the form of metal-exchanged sorbents (Maeck et al., 1968; Maeck and Pence 1970; Pence et al., 1970; Pence et al., 1972; Riley et al., 2017; Riley et al., 2020) or metal-impregnated sorbents (Matyáš et al., 2011; Matyáš et al. 2018; Matyáš et al. 2021b). Although they are often less kinetically favorable than iodine interactions with metals, metal-oxide composites (e.g., single metal oxides, spinel oxides, metal hydroxides) (Muhire et al., 2022) are also of interest as most realistic off-gas environments are highly oxidizing (Jubin et al., 2018). The oxidation state of the getter (e.g., Ag^0^ vs. Ag^+^) plays a vital role in the sorption performance due to differences in reactivity with components in the process stream. Metals with the ability to form oxyhalides, especially those with structural analogues of minerals, are favorable as they tend to have prolonged capture performance and stability for long-term storage (Tesfay Reda et al., 2021).

In regard to metals, those where the formation of iodides are thermodynamically favored over oxides are preferred due to the complexity of the process stream, a factor discussed below (Strachan et al., 2011). One such metal is silver, which has been extensively studied for the chemisorption of iodine from gases and aqueous environments for many decades (Jubin 1979). Studies examining alternative metals have typically incorporated them into oxide-based [e.g., zeolites (Maeck and Pence 1970; Pence et al., 1972), silica (Matyáš et al., 2011; Riley et al., 2017; Riley et al., 2020)] or carbon-based (Muhire et al., 2022) substrates. As the choices of substrate and getter loading method can affect iodine capture, a recent study was conducted using pure metallic substrates to eliminate these artifacts (Riley et al., 2021). This study demonstrated that silver, and other pure metals such as Al^0^, Cu^0^, In^0^, and Sn^0^ can be used to capture large quantities of iodine in saturated conditions at various temperatures. A variety of iodine mass loadings result based on the oxidation state of the metal upon reaction with I_2(g)_ to form the metal-iodide (MI_*x*_) complex, which for these metals is M^1+^ for Ag (AgI) and Cu (CuI), M^3+^ for Al (AlI_3_) and In (InI_3_), and M^4+^ for Sn (SnI_4_). The lower the molecular mass of the base metal and the higher the oxidation state of the metal in the MI_*x*_ complex, the higher the overall iodine mass loading in the MI_*x*_ complex (see Figure 2B for several examples).

### Kinetics of iodine adsorption

Understanding the kinetics of iodine adsorption is essential because the sorbent is required to scavenge maximum amounts of available iodine as fast as possible before it can escape the vicinity of the sorption environment (Figure 1B). In off-gas streams generated during the reprocessing of used nuclear fuel or the processing of legacy waste, this environment would include a large volume of carrier gas with a very low concentration of iodine (Riley et al., 2016). It should also be noted here that the species of iodine can vary from application to application, and could include a mixture of a variety of different iodine species in the same stream [e.g., I_2(g)_, ICl, CH_3_I] (Riley et al., 2016). Faster kinetics of adsorption means potentially less sorbent required to capture the same quantity of iodine (e.g., shorter bed depth) compared to sorbent with slower kinetics.

A comparison of the iodine sorption kinetics between silver and bismuth was conducted using nickel foam functionalized with either Ag^0^ (Ag-Ni) or Bi^0^ (Bi-Ni) (Tian et al., 2021). The study showed that Ag-Ni reached 90% of its saturation capacity in 30 min while 120 min was needed for the Bi-Ni. Although not specifically stated to contribute to the difference in sorption kinetics, significant differences were observed in the particle morphology after reacting with iodine. Iodine reactions with spherical silver particles led to the formation of larger AgI particles, while iodine interactions with rod-like bismuth particles led to the formation of needle-like structures. Changes in crystal structure and ability of iodine to penetrate through the oxide layer(s) present within the sorbents can also add to the time required for full reaction with the getter of choice.

### Performance in relevant process conditions

The performance of the getter can change drastically depending on the environment in which it is used to capture radioiodine (Figure 1C). In the reprocessing of used nuclear fuel, extremes in pH are observed depending on the process, such as acidic pH conditions [e.g., HNO_3(g)_, HF_(g)_] in off-gas streams or highly basic pH conditions in a caustic scrubber solution or a molten hydroxide scrubber (i.e., pH ≥ 12) (Riley et al., 2016). It is also an issue if the stability of the sorbent varies with temperature as some metals behave differently at low temperatures versus high temperatures (Riley et al., 2021). An additional point is that the performance of different getters can vary in terms of their abilities to scavenge low concentrations of the target species from a larger volume of competing species (e.g., Cl_2_) (Matyáš et al., 2021b). Finally, some of these environments can be highly oxidizing (e.g., NO_*x*_) which can cause unwanted reactions between free oxygen and the getter metals to oxidize active metal sites to metal oxides, inhibiting subsequent reactions with iodine (Backx et al., 1981; Campbell 1985; Bukhtiyarov et al., 1999; Matyáš et al., 2018).

Developing an understanding of iodine selection preference over other components present in the off-gas is critical as realistic conditions will contain competing components. The most obvious competitive species are other halogen-based compounds such as ICl and Cl_2(g)_. The chemisorption of Ag-based species for Cl_2(g)_ is not well studied but a recent study by Matyáš et al. (2021b) on the co-adsorption of Cl_2(g)_ and I_2(g)_ shows that the Ag^0^-functionalized silica aerogel can chemisorb both gases simultaneously and might have a preference of Cl_2(g)_ > I_2(g)_ based on this initial dataset. More work on these types of co-adsorption and competition experiments are needed for new sorbents so that the efficiency in real-world processing conditions can be predicted and evaluated accordingly.

Understanding sorbent performance in oxidizing environments is crucial as it will be the case in most of the known situations in which radioiodine capture is needed. An initial step in assessing oxidation potential is to conduct aging experiments, whereby candidate sorbents are subjected to relevant gas streams expected in the process stream for extended time periods and the sorbent performance is evaluated after these aging experiments. Aging experiments in 2% NO_2_ in dry air and 1% NO in N_2_ streams at 150 °C (for up to 6 months) were noted to drastically reduce the iodine capacity of reduced silver mordenite (Ag^0^Z) in a study by Wiechert et al. (2020), whereby Ag^0^ is oxidized to form molecular nitrates that are believed to be on the surfaces of the mordenite crystals. In their study, X-ray absorption spectroscopy was used to verify that a majority of the Ag present in the aged samples was in the oxidized (Ag^+^) state. The reactions shown in Eq. 1 (Backx et al., 1981; Campbell 1985; Bukhtiyarov et al., 1999), Eq. 2 (Bare et al., 1995), Eq. 3 (Outka et al., 1987; Zemlyanov et al., 1998), Eq. 4 (Carley et al., 1998), and Eq. 5 (Bao et al., 1999; Zemlyanov and Schlögl 2000) describe some of the mechanisms that are responsible for the effects observed in aged Ag^0^Z sorbents (note that “ads” refers to adsorbed species on the mordenite surfaces). Reactions between Ag^0^ and O_2_ or NO_*x*_ species can result in oxidized silver species, such as Ag_2_O, in the mordenite. Also, Eq. 4 shows how residual Na in the Ag^0^Z can act as a catalyst for dissociative adsorption of NO to convert Ag^0^ to Ag_2_O. Eq. 5 shows how NO can convert Ag_2_O to AgNO_3_ and Ag^0^ so while the oxidation states are shifting around during aging, the types and distribution of oxidized species can also shift. Thus, the surface chemistry of Ag^0^-containing species under different environmental conditions is complex.

$${4\text{ Ag}}^{\text{0}}\;+{\text{ O}}_{2}\;↔{\;2\;(\text{Ag}}_{2}{\text{O})}_{\text{ads}}$$ (1)

$${2\text{ Ag}}^{\text{0}}\;+{\text{ NO}}_{2}\;↔{\;(\text{Ag}}_{2}{\text{O})}_{\text{ads}}\;+\text{ NO}$$ (2)

$${\text{Ag}}_{2}{\text{O }+\text{ NO}}_{2}\;↔{\;(\text{AgNO}}_{3}{)}_{\text{ads}}{\;+\text{ Ag}}^{\text{0}}$$ (3)

$${\left(\text{Na}\right)}_{\text{ads}}{\;+\;2\text{ Ag}}^{\text{0}}\;+\text{ NO }\to {\;(\text{Na})}_{\text{ads}}{\;+\;(\text{Ag}}_{2}{\text{O})}_{\text{ads}}\;+\text{ 0}\text{.}5{\text{ N}}_{2}$$ (4)

$${{(\text{Ag}}_{2}\text{O})}_{\text{ads}}\;+\;2\text{ NO }\to {\;(\text{AgNO}}_{3}{)}_{\text{ads}}{\;+\text{ Ag}}^{\text{0}}\;+\text{ 0}{\text{.}5\text{ N}}_{2}$$ (5)

### Properties of the sorbent substrate

The physical properties of the substrate supporting the getter play a large role in iodine capture, including specific surface area (*SSA*), pore structure, and mechanical stability (Figure 1D). From a facility operation standpoint, permeable substrates are important to prevent a significant pressure drop in the sorbent containment chamber. The typical solid sorbent approach for high-flow applications is a packed bed of granular, pelletized, or other forms (e.g., berl saddles) of a moderately high *SSA* sorbent functionalized with a modest amount of getter. High-*SSA* substrates with an open cell structure tend to be more permeable and allow carrier gases or solutions to easily flow through the sorbent. Sorbents with low permeability can cause tortuous pathways for gas flow to move through the column and can lead to attrition and particulation of the sorbent bed (generation of fines), reducing the effectiveness of the sorbent. Commonly reported sorbents include porous alumina impregnated with AgNO_3_ (e.g., AC6120 from Clariant), Ag-KTC/KTB (AgNO_3_-loaded amorphous silicic acid) (Cheng et al., 2015), zeolites like mordenite (i.e., IONEX Ag-900) and Ag-faujasite (i.e., IONEX Ag-400), Ag^0^-functionalized silica aerogels (Matyáš et al., 2018), and Ag^0^-functionalized xerogels (Chong et al., 2021), which all have *SSA* values of ∼50–250 m^2^ g^−1^. Higher *SSA* values also provide more sites for the getter to enable higher iodine loading. However, the higher the *SSA* value of the sorbent, the less mechanically stable they tend to be under prototypical conditions (Matyáš et al., 2018). Friable materials have a greater potential to release particulates containing radioiodine into downstream unit operations.

The chemical nature of the substrate can lead to direct reactions with iodine. In a recent report on a carbon foam, both physisorbed I_2_ and chemisorbed tetraiodoethene (C_4_I_4_) were reported after exposure to iodine (Baskaran et al., 2022). These reactions were diminished as the foam was functionalized with increasing amounts of bismuth as chemisorption between the bismuth and iodine dominated. This finding opens the door to tuning the ratio of physisorption to chemisorption for enhanced capture of iodine from dilute gas streams.

Another aspect to consider is when the substrate has another redox-sensitive element present within its matrix. An example of this is the Ag^0^-functionalized silica aerogel developed by Matyáš et al. (2011; 2018) where the sorbent contains thiol (–SH) groups to help tether the Ag^0^ crystals to the aerogel surface using (3-mercaptopropyl)trimethoxysilane. The addition of these thiol groups changes the Ag^0^/Ag^+^ redox chemistry and drastically reduces the effects of aging compared to Ag^0^Z sorbents that were treated under the same aging conditions. This is because the thiol groups (S^[2-]^) oxidize to sulfate (S^[6+]^O_4_^2-^) during aging studies based on X-ray photoelectron spectroscopy data thereby reducing the impact on the Ag^0^ → Ag^+^ reactions (Matyáš et al., 2018).

### Environmental stability and disposition pathways for iodine-loaded materials

The environmental stability of the metal iodide formed after capture considers the chemical surface reactivity and morphological changes that could occur during operation and upon long-term storage after capture (Figure 1E). Some examples of where this is important were documented in a recent paper by Riley et al. (2021) where some of the highest performing pure metals for I_2(g)_ capture were indium (forming InI_3_) and tin (forming SnI_4_). While In and Sn metals have a tremendous affinity for I_2(g)_ forming a triiodide and tetraiodide (i.e., 76.8 and 81.0 mass% iodine, shown in Figure 2B), respectively, neither MI_*x*_ complex is environmentally stable. The SnI_4_ complex is not a very chemically durable species and InI_3_ is extremely hygroscopic leading to deliquescence in moisture-containing air (Riley et al., 2021). Oxyhalides, such as BiOI, are attractive from a stability standpoint; however, their formation can be complicated because they form as metastable BiI_3_ (that converts to BiOI) and reactions with Bi_2_O_3_ might not be thermodynamically favored. Therefore, iodine is initially release as BiOI forms, so extra Bi^0^ would need to be added to a waste form if this phase was formed to capture the evolving I_2_ during the phase transition—see Eq. 6. These are the types of parameters that need to be considered when designing next-generation sorbents for real-world applications.

$${2\text{ BiI}}_{3\left(\text{s}\right)}{\;+\text{ O}}_{2\left(\text{g}\right)}\;\to {\;2\text{ BiOI }+\;2\text{ I}}_{2\left(\text{g}\right)}$$ (6)

The disposition pathway for the iodine-loaded material is very important as it has to meet repository requirements if disposal is the planned pathway. The disposition pathway of the iodine-loaded sorbent is dictated by stability of the iodine-containing compound during transformation of the sorbent into a waste form and the chemical durability of the waste form. Sorbent transformation into a waste form is performed to condense the material into a smaller volume and this prevents potentially friable material from being released into the environment. Volatilization of iodine during the processing to a waste form can be mitigated through low-temperature consolidation (Garino et al., 2011; Sava et al., 2012; Garino et al., 2016), spark plasma sintering (Le Gallet et al., 2010; Yao et al., 2014), or hot-isostatic pressing (Bruffey and Jubin 2015; Maddrell et al., 2015; Maddrell et al., 2019). Once synthesized, the resulting waste form undergoes a variety of testing protocols to determine its resistance to attack by atmospheric agents or aqueous solutions that simulate different potential repository conditions. These tests are often conducted at elevated temperatures and pressures to accelerate the leaching effects and predict material chemical durability over geological timescales.

### Other considerations

In addition to the selection criteria provided above, other aspects exist that should be considered. One of the more important ones is the option of recycling the sorbent to enable reuse for future capture. For physisorption-based sorbents, this could involve iodine desorption through an elevated temperature process. For chemisorption-based sorbents, recycling would be a far more involved process whereby the iodine has to be chemically stripped from the getter by means of decomposition (e.g., through high-temperature treatment) or substitution reactions (e.g., through chemical means). For example, iodine from AgI can be driven into solutions using a sodium sulfide reaction to convert Ag in AgI to Ag_2_S (solid precipitate) whereby I is soluble as I^−^ and Na^+^ remain in solution (Cao et al., 2017). It is possible that Ag_2_S could be recycled into future sorbents. Recycling of chemisorption-based sorbents would be very valuable from both economic and environmental perspectives because it would prevent expending limited precious metal resources.

The second aspect mentioned is the development and understanding of adsorption isotherm models. These types of models help researchers understand how and why particular species adsorb to substrates. There are six main types of experimentally observed adsorption isotherms, which can be used to describe both physisorption and chemisorption (Keller 2005). In realistic environments (i.e., mixed gases), no single isotherm can describe all of the adsorption phenomena in these systems. It is especially challenging for iodine as the concentration is extremely low compared to other species in representative off-gas streams. Regardless, adsorption isotherms can provide to guide the design and performance of future sorbents. Empirical models are needed at this time because the complexity of interactions between the adsorbing molecules and the sorbent hinder accurate predictions using theoretical models (e.g., statistical molecular methods).

### Future perspectives

The majority of research on sorbents for iodine capture has looked at materials under idealistic and nonrepresentative conditions (Criterion 1 and Criterion 2). This fundamental base understanding of materials performance has provided us with information that was used to down select materials for investigation in environments with oxidants and/or competing species (Criterion 3). Studies on the role of the sorbent will are becoming more dominant as we learn that it can be used to use the properties to “protect” the getter from unwanted interactions or enhance capture performance through physisorption (Criterion 4). The final fate of the iodine-loaded material is of utmost importance, so the chemical durability and environmental stability need to be considered (Criterion 5). Studies that combine all the components of prototypical off-gas streams will be necessary for each application. A major aspect when evaluating stability of the sorbent and the final waste form is radiation stability, as both iodine-129 and iodine-131 decay to xenon. Many of the candidate sorbents in discussions today have not been evaluated under relevant radiological conditions to assess their stabilities within these environments. These unknowns provide a lot of exciting and important areas for future researchers. While this list does provide some of the more important criteria for consideration, it is by no means a comprehensive list and readers are referred to reviews on this topic for more comprehensive criteria and other aspects for sorbent evaluation.

## Data Availability

The original contributions in presented in this study are included in the article, further inquiries can be direct to the corresponding author.
